# Techniques for Evaluating the Fit of Removable and Fixed Prosthesis

**DOI:** 10.5402/2011/348372

**Published:** 2011-07-14

**Authors:** Mallika S. Shetty, K. Kamalakanth Shenoy

**Affiliations:** Department of Prosthodontics, Yenepoya Dental College, Deralakatte, Mangalore, Karnataka 575018, India

## Abstract

The importance of an accurately fitting fixed prosthesis or a removable prosthesis is essential for the success of the restoration. Ill-fitting prosthesis may cause mechanical failures of the prosthesis, implant systems, or biologic complications of the surrounding tissue. There are several causes related to improper seating of the prosthesis. Some of which can be corrected and the others need to be repeated. Hence the clinician must carefully evaluate the adaptation of the prosthesis using the clinical techniques and combination of the available materials and evaluation methods to optimize the fit of prosthesis. This article reviews the various clinical methods that have been suggested for evaluating the fit of the fixed and removable prosthesis.

## 1. Introduction

An accurately fitting fixed prosthesis on the prepared tooth or a removable prosthesis on the denture bearing area is of paramount importance for the success of the restoration [1]. Achieving a passive fit between a cast metal framework or bar and the supporting implant abutment is essential for long-term success of an implant-supported restoration [2]. Ill-fitting prosthesis may cause mechanical failures of the prosthesis, implant systems, or biologic complications of the surrounding tissue.

The purpose of this paper is to review the various clinical methods that have been suggested for evaluating the fit of the fixed and removable prosthesis.

**Several causes can be related to the improper seating of casting:**

1. improper line of draw with adjacent teeth,
2. undercut in the preparation,
3. distorted impression,
4. abraded dies,
5. overextended wax patterns,
6. distorted wax,
7. improper expansion of the investment,
8. improper burn-out technique,
9. nodules on the casting,
10. distorted casting,
11. excessive proximal contacts [1].

**Causes that can be related to poor fit of removable prosthesis are as follows:**

1. distorted impression,
2. improper block out and waxing,
3. processing errors,
4. improper metal or acrylic finishing and polishing.

**Factors affecting the fit of implant prosthetic superstructure are as follows:**

implant alignment,
impression technique and materials,
stone expansion,
wax distortion,
investment expansion,
metal shrinkage,
acrylic/porcelain shrinkage,
manufacturer variance/tolerance component variance, analog variance [3],
design configuration clinician and technician experience [4],
failure to create an accurate working cast,
the transfer technique [2].

Various materials and techniques have been suggested to disclose discrepancies of fit of implant framework, FPD, and RPDs.

## 2. To Disclose Discrepancies of Fit between the Casting and the Prepared Tooth, the Materials That Can Be Used Are the Following

Inspect the internal surface of the restoration under magnification, for small nodules of metal or residual investment. Remove the metal nodules with a half-round high-speed bur.

### 2.1. Disclosing Wax

Fill the restoration with disclosing wax, and heat it over the flame until wax flows, so that it flows into the pores of the metal and adhere to the internal surface of the restoration. Allow the restoration to cool before try in. Then place the restoration on the tooth and seat it. Test the interproximal contact with floss, and adjust if excessive. Repeat the process until the interproximal contacts are perfected.

Next seat the restoration with firm pressure and then remove and inspect the internal surface. Any area which keeps the restoration from seating will appear as a bright shiny spot. Adjust that area with a half-round high-speed bur. New wax is added before another trial on the tooth. This process should be repeated until the wax on the inner occlusal surface becomes very thin. Warm the restoration and remove the wax before cementation [5, 6].

### 2.2. Chloroform and Rouge

Chloroform, a potent solvent, dissolves the rouge. Chloroform and rouge mixture is painted on the intaglio surface of the cast restoration. Chloroform quickly evaporates and leaves a thin film of rouge that helps detect areas of interference as during the fitting of a restoration. Before clinical try in, care must be taken to ensure that the chloroform has completely evaporated and has not pooled on the restoration surface, as chloroform is known to be a potent skin and mucous membrane irritant. Chloroform is also hepatotoxic and nephrotoxic and may be fatal if swallowed, inhaled, or absorbed through skin [7, 8].

Halothane serves as an excellent alternative to chloroform. It is relatively nontoxic. The vapors of halothane are nonirritating to the respiratory tract. Environmentally, halothane is considered much safer than chloroform. When used a disclosing medium, halothane rapidly dissolves rouge and forms a homogenous solution. High spots and internal discrepancies are easily detected with this medium and because of its thin consistency; further applications of halothane and rouge do not form layers or result in excessive film thickness. It is easily cleaned from the casting with steam or aluminium oxide abrasive [7].

### 2.3. Polyvinyl Siloxane Impression Material, Low Viscosity Type 1

Place the material in the restoration, and seat it on the prepared tooth. Have the patient exert biting pressure on the restoration. Do not remove excess material from margins. When the restoration is removed from the mouth, note perforations in the material and mark these spots with red pencil on the casting. Remove the polyvinyl siloxane impression material from the casting and adjust the red marks in the casting with a high-speed hand-piece and a carbide bur. Repeat the above until the material does not show through the impression material and there is a uniform thickness of impression material inside the restoration [9]. 

## 3. To Evaluate the Adaptation of the Removable Prosthesis, the Following Are the Disclosing Materials Recommended

Materials are as follows:

disclosing wax,
occlude disclosing medium,
pressure indicating paste,
fit checking sprays,
chloroform and rouge,
polyvinyl siloxanes.

To use disclosing media effectively, dry off the framework in the areas where the disclosing medium will be applied. Apply the disclosing medium, gently seat the framework, and remove. Areas that exhibit metal showing through should be adjusted. The old disclosing medium should be cleaned off, new medium applied, and the framework tried in again. One should avoid overreducing contacts on guideplanes, rests, and retentive tips. Contacts between the framework and the teeth below the survey line should not be arbitrarily removed, because these contacts can help guide the framework into place and provide some degree of retention and stability. With sufficient practice, the dentist should be able to distinguish between marks caused by interferences and those resulting from rub-off as the framework is inserted and removed [8, 10, 11].

The advantages of disclosing waxes are that it provides three-dimensional representation of framework adaptation and it shows the degree of interference.

The disadvantages of disclosing wax are that it requires a flame source and is relatively difficult to remove [10].

The advantages of polyvinyl siloxanes are that they are easy to read and remove from the framework, provide a three-dimensional perspective, and have minimal thickness.

The disadvantages of polyvinyl siloxanes are that they are expensive, require mixing, and need time for the material to set [10, 12].

The advantages of occlude disclosing medium are that it marks areas of interference well and is easy to clean off. 

The disadvantages of occlude medium are that it is expensive, there is potential for applying too thick layer of material, and it is difficult to work with in a wet environment [10].

The advantages of chloroform and rouge are that it is easy to apply and identifies interferences well.

The disadvantages of chloroform and rouge are that it is difficult to remove and it has carcinogenic potential.

## 4. In Case of Complete Dentures, the Techniques Used to Adjust Areas of Irritation Involve the Following

**Direct Visualization**

This technique borders on guesswork about where and how much acrylic needs to be removed.

**Pressure Indicating Paste**

Frequently gives results that are hard to interpret, and it is also messy and time consuming to clean from deeply fissured denture base.

**Color Transfer Applicators**

Rely on sore spots being easily visible and do not provide guidance on the extent of the necessary adjustment.

**Fast Setting Irreversible Hydrocolloid Material (Alginate)**

Mix a small amount of impression material. Reline the denture in the area in question. Seat the prosthesis in the mouth and have the patient close into normal occlusion. Allow the material to set and then remove the denture carefully without tearing the alginate. The area needing adjustment usually is easy to visualize. The denture is relieved, and the alginate can be simply peeled away. The process is repeated until no high spot is seen [13].

**Vinyl Polysiloxane Impression Material**

Mix Vinyl polysiloxane impression material, and reline the denture. After the impression material sets, evaluate it to check the fit of the denture base to the basal tissues and peel it out of the denture. Little or no cleanup is required [14].

**Zinc Oxide Base Paste**

Apply Zinc oxide base paste on the intaglio surface of the denture. Seat the prosthesis in the mouth and have the patient close into normal occlusion. Then remove the denture. The area needing adjustment is easily visible. The denture is relieved. This procedure is repeated until no high spot is seen. Cleanup of denture is then done.

## 5. Methods for Evaluating Fit of Implant Prosthetic Superstructure

**(1) Alternate Finger Pressure**

Manually seat the prosthesis with finger pressure, applying pressure alternately over one terminal abutment and then the other. Finger pressure applied across the arch of the framework can be used to check for lift or distortion. Any detected rocking or saliva movements between the framework abutment interface is considered a misfit [15].

**(2) Direct Vision and Tactile Sensation**

Direct vision in conjunction with tactile sensation with an explorer is a method commonly used to evaluate the implant framework fit. This method can be enhanced when used with ample lighting and magnification [15, 16].

**(3) Radiographs**

Periapical radiographs are often used to evaluate framework fit [15].

**(4) One Screw Test**

It is recommended to tighten one screw at one terminal abutment and discrepancies observed at the other abutments. This technique is effective for long span frameworks. The one screw test can be used in conjunction with direct vision and explorer when the margins are supragingival or with periapical radiographs when the margins are subgingival [15].

**(5) Screw Resistance Test**

In this method gold screws are tightened one by one, starting with the implant closest to the midline until initial resistance between the head of the screw and the framework is encountered; a final 180 degree turn is performed to reach a torque of 10 Ncm for complete screw seating. If more than a half turn is needed to provide seating of the gold screw, the framework is a misfit. The presence of persistent pain, pressure, and discomfort during the tightening of the screws may also indicate an unacceptable level of framework misfit [15].

**(6) Disclosing Media**

Disclosing media were used to evaluate the fit of the framework on the implant abutments in the same manner as are used to ensure complete seating and passivity for conventional fixed and removable partial dentures.

Fit Checker, pressure indicating paste, and disclosing wax have been used for evaluation of framework fit.

**(7) Materials Like Unwaxed Floss**

Polyester film strips and Shim stock are also suggested as tools to verify framework fit [15].

Any discrepancy in fit demands framework sectioning, solder indexing, soldering, and then a clinical evaluation of the fit [17, 18].

## 6. Summary

The success of prosthesis depends on how well it fits without causing injury to the remaining teeth and soft tissues.

There are several causes related to improper seating of the prosthesis. Some of which can be corrected, and the others need to be repeated.

Improving the clinical techniques and combination of the available materials and evaluation methods can optimize the fit of prosthesis.
